# Identification and functional characterization of a novel homozygous intronic variant in the fumarylacetoacetate hydrolase gene in a Chinese patient with tyrosinemia type 1

**DOI:** 10.1186/s12920-022-01406-6

**Published:** 2022-12-03

**Authors:** Jiao Chen, Junhui Sun, Xuefang Li, Mengmeng Du

**Affiliations:** 1 Department of Medical Genetics and Prenatal Diagnosis, Taizhou People’s Hospital, Taizhou, China; 2 Reproductive Medicine Center, The First Affiliated Hospital of Wenzhou Medical University, Wenzhou, China

**Keywords:** Hereditary tyrosinemia type 1, Cryptic splice site variant, Alternative transcripts, Functional analysis, *FAH* gene

## Abstract

**Background:**

Hereditary tyrosinemia type 1 (HT1; OMIM# 276700) is a genetic metabolism disorder caused by disease-causing variants in the fumarylacetoacetate hydrolase (*FAH*) gene encoding the last enzyme of the tyrosine catabolic pathway. Herein, we describe the clinical features and genetic characteristics of HT1 in a five years and seven months old Chinese patient.

**Methods:**

After clinical diagnosis of the proband with HT1, genetic testing was performed by Sanger sequencing of the *FAH* gene in all family members. Functional analysis of the disease-causing variant was performed by cDNA sequencing to understand the effect of the variant on *FAH* transcript. To further predict the variant effect, we used Human Splicing Finder (HSF) and PyMol in silico analysis.

**Results:**

We identified a novel previously undescribed intronic variant in the *FAH* gene (c.914-1G>A). It was detected in a child who was homozygous for the variant and had the clinical presentation of HT1. cDNA sequencing showed that this splice-junction variant affected the transcription of *FAH* by formation of two different transcripts. Our observations and laboratory experiments were in line with in silico methods.

**Conclusions:**

Our study provides new insight into the HT1 variant spectrum and a better understanding of this disease in the Chinese population. This will be useful for molecular diagnosis in our country in cases where premarital screening, prenatal diagnosis and preimplantation genetic diagnosis are planned.

## Introduction

Hereditary tyrosinemia type 1 (HT1; OMIM# 276700) is an autosomal recessive disorder caused by deficiency of fumarylacetoacetate hydrolase (FAH), the last enzyme of tyrosine degradation pathway [1, 2]. Insufficient activity of FAH leads to the accumulation of toxic metabolites such as succinylacetone (SA), which is a pathognomonic finding [3]. These metabolites disrupt the cellular metabolism in various body tissues, predominantly in liver, kidneys and central nervous system [2]. Most patients present with progressive liver disease with increased risk of hepatocellular carcinoma, a secondary renal tubular dysfunction and porphyria-like syndrome [4]. The incidence of HT1 is approximately 1/100,000 [5].

At present, the effective treatment for HT1 is using Nitisinone (NTBC), a drug which inhibits parahydroxyphenylpyruvic acid dioxygenase (HPPD), the second step in the tyrosine degradation pathway, thereby preventing the accumulation of fumarylacetoacetate and its conversion to succinyl acetoacetate and SA [6, 7]. In other way, liver transplantation is considered another curative option at a liver failure in an undiagnosed patient, at a NTBC treatment failure and at a hepatocellular carcinoma emergence [8].

The FAH enzyme, mainly expressed in liver and kidneys [9], is encoded by *FAH* gene located on chromosome 15 (15q25.1) and consisting of 14 exons spanning over 35 kilobase (kb) of DNA [10, 11]. So far, close to 100 variants in the *FAH* gene have been associated with HT1 [5]. Exons 9 and 12 have the largest clusters of HT1 disease-causing *FAH* variants. Interestingly, both exons contain metal and substrate binding sites [12]. In this report, we present the identification process and functional characterization of a novel intronic splice-junction variant in the *FAH* gene in a Chinese patient with HT1. We investigated the cDNA pattern of the patient in an attempt to gain insight into the novel splice variant effect as a main cause of HT1. Taking the intronic variant of *FAH* gene into consideration is strongly suggested in genetic counseling and etiology research for HT1.

## Materials and methods

### Patient

A 5 years and seven months old girl who had suffered from liver disease came to the Department of Medical Genetics and Prenatal Diagnosis, Taizhou People’s Hospital, China for clinical diagnosis and genetic counseling. Data related to the pedigree of the patient were collected from her parents. The clinical assessment was performed, including physical and ultrasonic examinations. We also measured some biochemical parameters. The proband’s parents and her 2 years old younger brother were unaffected and did not have clinical signs of liver disease. We did not obtain medical history from her family members. The project was approved by the Ethics Committee of Taizhou People’s Hospital and all described procedures were complied with the Helsinki Declaration. A signed informed consent was obtained from the proband’s parents.

### Genetic testing

Whole blood (2 ml) was collected from the affected proband, her parents and her younger brother. Genomic DNA was extracted from peripheral blood leukocytes using the Blood Genomic extraction kit (Tiangen Biotech, China). We designed 14 pairs of primers (Table 1) that could cover all exons and their exon-intron boundaries of *FAH* based on the reference sequence (NC_000015.10) using standard polymerase chain reaction (PCR) followed by Sanger sequencing. Human Splicing Finder (HSF) (http://www.umd.be/HSF/) was used to predict the influence of the variant to mRNA splicing.

**Table 1:** Primer sequences used to analyze the exons and the cDNA of *FAH* gene

Primer name	Primer sequence (F)	Primer sequence (R)	Product size (bp)
*FAH*-exon 1	CGGTGAGACCAAAAGTCAGGT	GCAAACCTGCGGACAATGAG	478
*FAH*-exon 2	ACCGCACAACTGAACTACCC	GTTGGCATCCACAGTAAGTGC	590
*FAH*-exon 3	TGGAGTGTGCCTCTACTGGA	CCGTGACTCAGAATGGCACT	533
*FAH*-exon 4	ATTCTGAGTCACGGCTTGGC	ATTCTGAGTCACGGCTTGGC	590
*FAH*-exon 5	CACAGGGACAAGGGAGAAGTC	GAACTTGGCAGCTCCTGAGAC	557
*FAH*-exon 6&7	AGCTCTGATGCCCTGCATT	CTGCCGATGTGGCTGAAGAG	423
*FAH*-exon 8	CACCACTGCACTCTGCTTTC	TCACCTGCCACTTTTGACCT	400
*FAH*-exon 9	GGACCTCTGTCCTTGGCATT	GAGCTTCCCTCCTGATGGTCT	420
*FAH*-exon 10	TCCTGCTGTCTCAGACCCTC	AGACGAGCCACACACATCTC	336
*FAH*-exon 11	GTGGGAGGAGGAAGTGATGA	AAAAGAGACAGGCCATGGAA	402
*FAH*-exon 12	AAGGGACTGGAGAGAGCTGG	GTGAGATACACCCACCTCGG	372
*FAH*-exon 13	TGGTGCATGTGTCACTCACT	AGCTAGAACAGTGCATGGCG	583
*FAH*-exon 14	GCTTCCGTGGAGGGTTATTCT	AGGAGGCCTGGGATGTCTAAT	539
*FAH*-cDNA-1	GTCCTTCATCCCGGTGGC	GGTAGCCCACTGGTAAGTGC	476
*FAH*-cDNA-2	CACCTTCCAGCCACCATAGG	TCGTCATGGCACAGATACGG	527
*FAH*-cDNA-3	TGGGAGTATGTCCCTCTCGG	TCATGATGGCAGGAGAGCAG	537

### cDNA analysis

Total RNA was extracted from the peripheral blood of the proband using Trizol Reagent (Takara) and reverse transcription polymerase chain reaction (RT-PCR) was performed to get the cDNA using PrimeScript™ RT Reagent Kit (Takara). Then we used 3 pairs of designed specific primers (Table 1) to amplify the *FAH* cDNA. The RT-PCR products were subjected to Sanger sequencing.

### cDNA sub (TA) cloning

In order to confirm the cDNA sequencing result and to understand the effect of the splice-junction variant on the mRNA splicing, cDNA PCR products were cloned into the pMD19-T vector (Takara, Dalian, China). Sanger sequencing was followed using universal primer M13.

### 3D structure model assay

In order to study the effect of c.914-1G>A on three-dimensional (3D) crystal structure of the protein, computational modeling was performed for normal and mutant FAH molecules using online software Swiss-model (https://swissmodel.expasy.org/). The template protein structure was taken from the Protein Data Bank (PDB) (https://www.rcsb.org/pdb/home/home.do) (Identified ID:1HYO) and the visual illustration was displayed by Pymol software.

## Results

### Case presentation

**Fig. 1:**
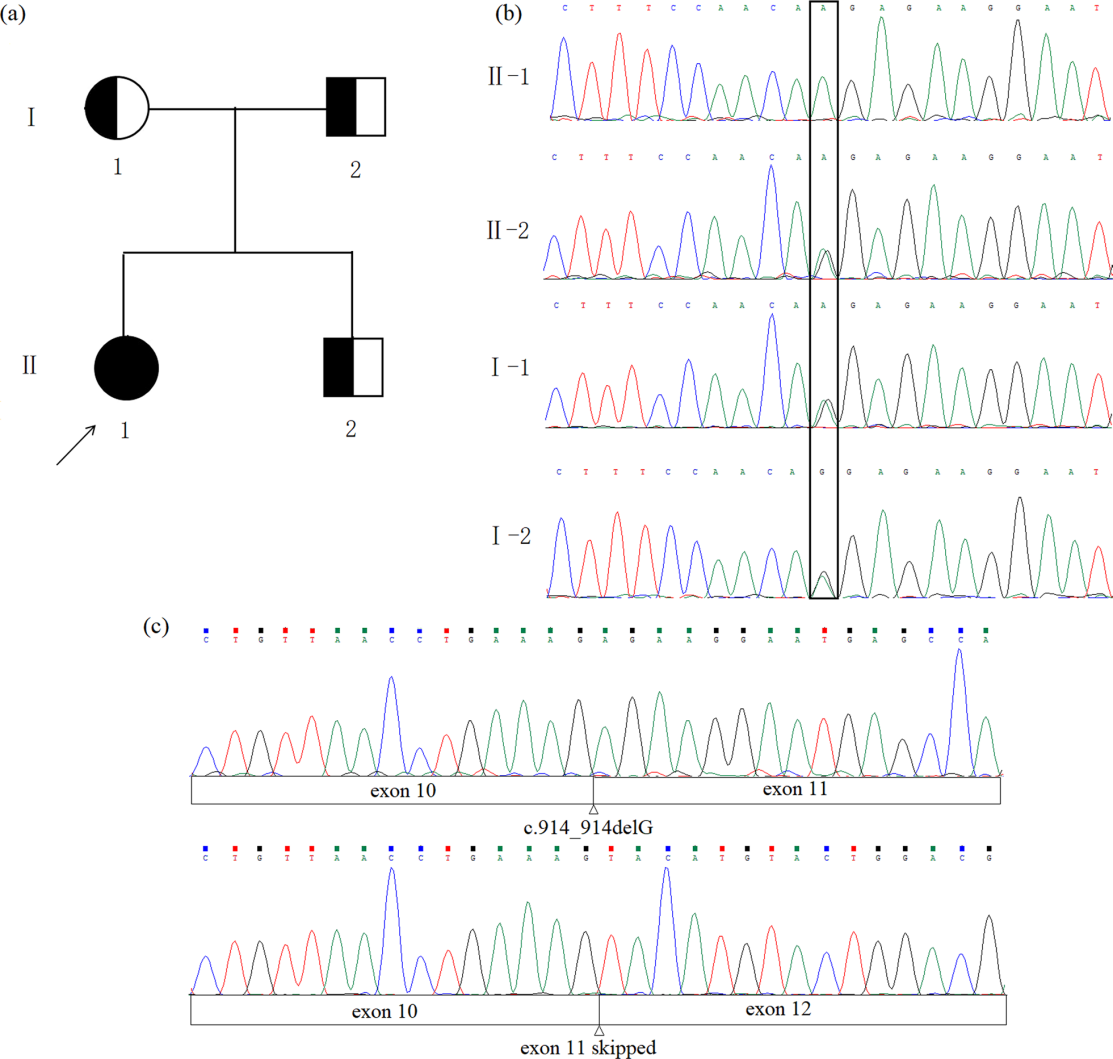
Pedigree and the *FAH* sequencing results **a** pedigree of the family. Half-filled symbols represented the variant carrier; Arrow indicated the proband. **b** The sequencing results of the family. The rectangular symbol indicated the variant site. **c** Illustrations of the proband sequencing results of cDNA TA cloning products. The top one indicated the c.914-1G>A led to a 1 bp deletion; the below one indicated the c.914-1G>A led to skipping of the entire exon 11

The proband (II-1, Fig. 1a) was a five years and seven months old girl from China and the first child of her parents. On physical examination, the patient looked sick, pale and had shorter stature. Abdominal ultrasound examination indicated hepatic cirrhosis, splenomegaly and abdominal flatulence which were confirmed by Computed tomography. The patient’s liver function tests showed a serious liver failure, alanine transaminase (ALT): 51 U/L (Reference Interval (RI): 7–40), aspartate transaminase (AST): 80 U/L (RI: 13–35) and gamma glutamyl transferase (GGT): 126 U/L (RI: 7–45). The result of AFP was 31283 ng/ml (RI: 0.89–8.78). Blood tyrosine, phenylalanine and methionine levels were 426 µmol/L (RI: 20–110), 157 µmol/L (RI: 30–75) and 283 µmol/L (RI: 9–40), respectively. Blood examination for organic acids showed elevated SA level with 6.6 µmol/L (RI: <1 µmol/L).

### Molecular diagnosis

PCR-based Sanger sequencing in both the forward and reverse direction of coding regions and exon-intron boundaries of the *FAH* gene from the proband (II-1), her parents (I-1 & I-2) and her younger brother (II-2) revealed that the proband carried a novel homozygous point variant c.914-1G>A of the *FAH* gene. Her parents and younger brother were heterozygous for the same variant. (Fig. 1a, b).

### Functional analysis

In order to evaluate the impact of this variant, HSF was applied to analyze the adjacent DNA sequence [13]. It suggested that the variant probably abolished the wild type splicing acceptor site (c.914-1) and induced an alternative splicing site at c.914 (Table 2).

**Table 2:** Analysis of native and potential alternative splicing elements by HSF

Position	Type	Motif	Value (0–100)
c.914-1 (WT)	Splice acceptor	tctttccaacagGA	92.66
c.914-1 (mutant)	Splice acceptor	tctttccaacaaGA	Site broken
c.914 (potential)	Splice acceptor	ctttccaacaagAG	78.65

Sequencing of the patient’s *FAH* cDNA showed that most of it was normal except the exon 10–12 (Data not shown). To confirm the elusive cDNA sequencing result, sequencing of cDNA TA cloning products showed that this novel homozygous point variant could lead to aberrant splicing followed by formation of two different *FAH* transcripts. One induced an alternative splicing site leading to loss of one nucleotide consistent with the prediction of HSF and the other caused skipping of the entire exon 11 of *FAH* (Fig. 1c). Sometimes the first transcript was produced and sometimes the other.

**Fig. 2:**
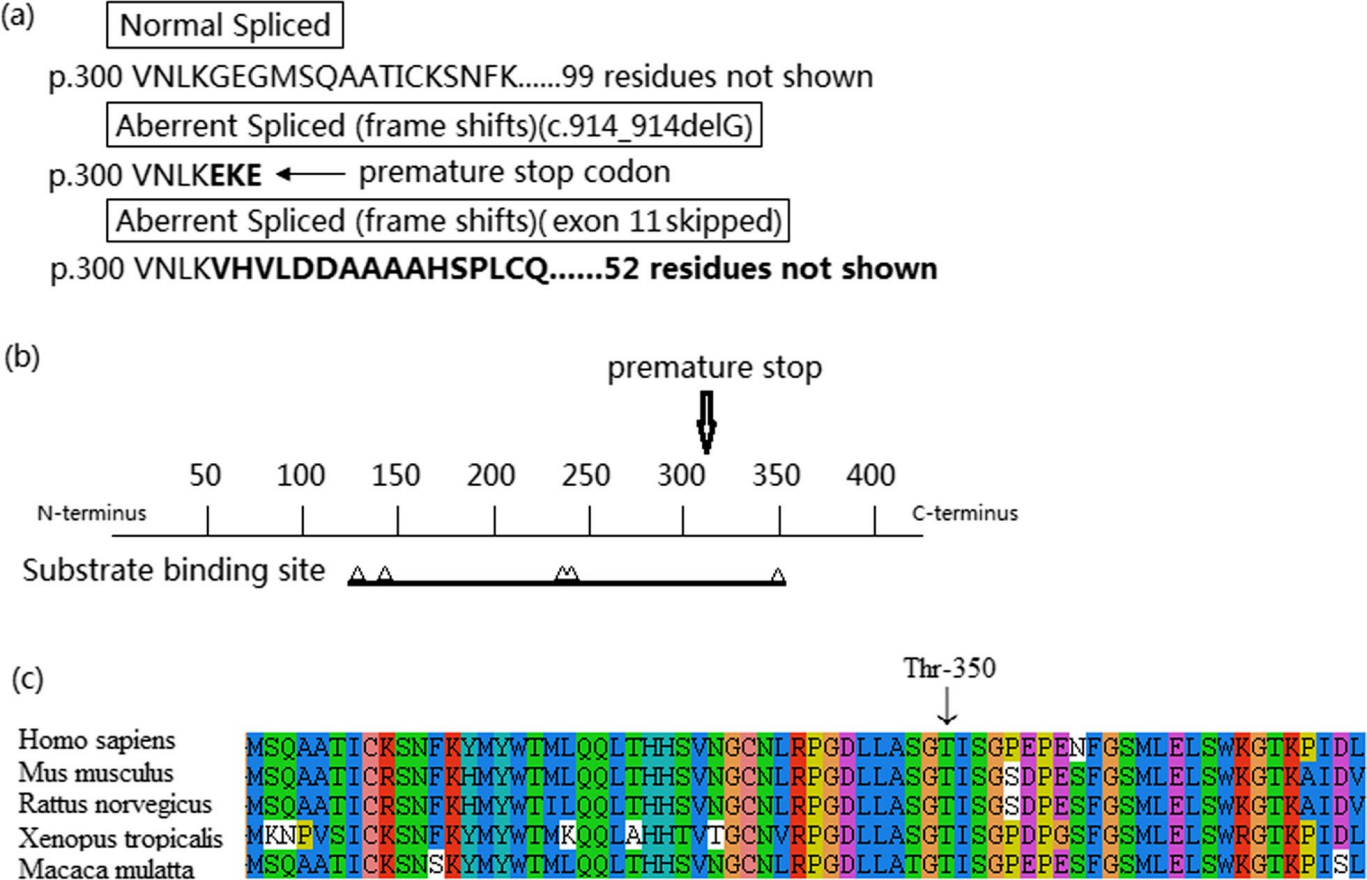
Prediction of the functional influence of *FAH* variant c.914-1G>A. **a** The predicted amino acid sequences of normal and mutant transcripts. The variant produced a truncated FAH protein and another different protein. **b** The position of potential premature stop codon of the truncated protein and associated functional domain. The numbers indicated the residue number. One substrate binding site was damaged due to premature stop codon. **c** Multiple-sequence alignment of FAH from different species revealed that Thr 350, one of the five substrate binding sites, was located within a highly conserved region

**Fig. 3:**
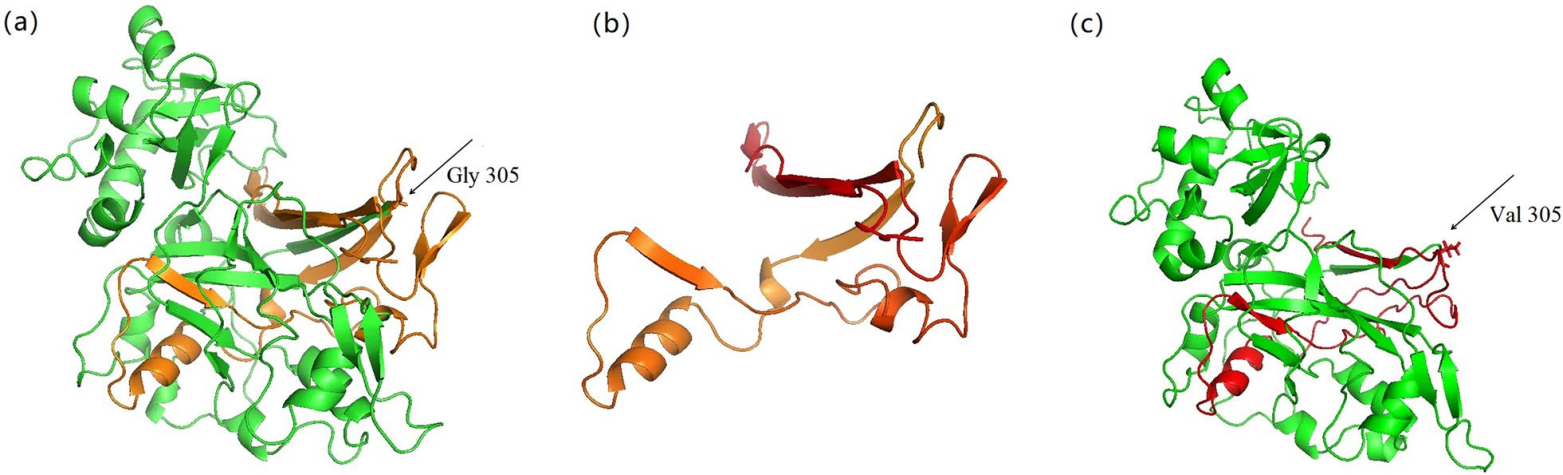
The 3D structures of wildtype and mutant FAH proteins. **a** 3D modeling of the wildtype FAH protein using PyMol software. The structure of 1–304 amino acids was shown as green and the structure of 305–419 amino acids was shown as orange. The arrow indicated the position Gly 305. **b** 3D modeling of the structure of 305 to 419 amino acids which was lost by the truncated FAH protein (c.914_914delG) in comparison with the wildtype protein caused by the frame-shift and premature termination. **c** 3D modeling of the mutated protein (exon 11 skipped) which lost a lot of crucial amino acids and the structure was different from Val 305 as indicated by the arrow. The red part was the changed structure in comparison with the wildtype protein

The loss of one nucleotide in the patient’s *FAH* transcript resulted in a stop codon, leaving 112 amino acids truncated. In the other transcript, entire exon 11 was skipped leading to frame-shift and premature termination (Fig. 2a). The 3D structure prediction was then performed of the two mutated proteins respectively using online software Swiss model. We depicted the schematic diagram of the FAH protein shown as the primary model (Fig. 3a), truncated model (Fig. 3b) and mutated model (Fig. 3c). The amino acid sequence was different when compared with wildtype sequence, and one protein was truncated (Fig. 3b) and the other lost part of the important strands (Fig. 3c).

## Discussion

HT1 can lead to severe liver and renal dysfunction [14]. Clinical and biochemical findings of the patient indicated that she suffered from HT1, which was caused by variants of *FAH* gene. The *FAH* gene contains 14 exons and encodes a 419 amino acid protein. In this study, we identified a novel splice site variant in the *FAH* gene (c.914-1G>A) in intron 11 which had not been previously reported. Typically, genome research focuses more on exon variants to identify disease-causing variants. This type of research is usually performed by whole exon sequencing. Geneticists and physicians usually overlook intronic variants which can be confirmed by in vivo or in vitro assays [15]. When a genetic testing of the coding regions of the *FAH* gene does not confirm the diagnosis, further investigation of intronic (non-coding) regions is needed [16]. There are about 18 intronic disease-causing variants reported within the *FAH* gene [5]. The most frequent HT1 variant encountered is the c.1062+5G>A (IVS12+5G>A) splice variant [17–19]. The c.554-1G>T (IVS6-1G>T) splice variant is also frequently identified, showing a high prevalence in the Mediterranean region and in southern Europe [19, 20].

The variant c.914-1G>A was located on the −1 bp site of the exon/intron junction. Usually, −3 to +6 positions of the exon/intron junction are considered highly conserved nucleotide sites. Variants in this area may affect splicing to various degrees by changing the consensus sequence, splicing enhancers, or silencers [21, 22]. In fact, the preferred way to study the splice abnormalities in a transcript is to focus on the fresh sample of the affected patients because the real splicing process is quite complicated in vivo. In this study, we found two different transcripts caused by the novel splice variant in vivo assay.

In order to predict the possible deleterious effects of the novel pathogenic variant, we performed in silico modeling based on the crystallographic structure of mouse FAH, which has 89% sequence identity with human FAH at the amino acid level (PDB Code: 1HYO). The 3D-modeling result provides explicit evidence of the influence of the variant. There are five substrate binding sites in the protein structure (Fig. 2b) which may combine together to form a hook-like conformation to bind the substrate. The truncated FAH lost a lot of crucial amino acids including an evolutionarily conserved amino acid Thr 350 (Fig. 2c), which was one of the five substrate binding sites. It probably affected the protein stability and enzyme activity.

About the genotype-phenotype correlation, in previous study, heterogeneous phenotypic patterns were observed in 13 patients with the homozygous variant c.554-1G>T (IVS6-1G>T) of *FAH* gene, ranging from a chronic renal phenotype without important hepatic symptoms to acute liver failure in the first months. Moreover, no phenotype differences were observed in the patients when compared with the patients of compound heterozygosity for the c.554-1G>T (IVS6-1G>T) and a stop mutation in the other allele [23]. Patients with the same genotype presented with various clinical manifestations, indicating that epigenetic or environmental factors could play an important role in modulating the phenotype in HT1 [24]. HT1 can present with liver disease at any age. The spectrum of liver disease ranges from an acute liver failure to hepatic cirrhosis and hepatocellular carcinoma [25]. Increased SA in blood provided the key for diagnosis. In our study, the patient had no symptoms of liver disease throughout infancy, showing chronic form with progressive hepatic disease.

## Conclusions

In summary, we diagnosed a Chinese patient with HT1 using biochemical and molecular analysis, revealing a novel homozygous splice-junction *FAH* variant (c.914-1G>A) which caused aberrant splicing followed by formation of two alternative transcripts. This identification will be of use for molecular diagnosis in our country to prevent this disease in the aspect of future carrier testing, prenatal testing, premarital screening and preimplantation genetic diagnosis.

## Data Availability

The detected variant has been submitted to the LOVD, direct link: https://databases.lovd.nl/shared/individuals/00419319. The other web links of the relevant datasets were as follows: Swiss-model (https://swissmodel.expasy.org/), Protein Data Bank (PDB) (https://www.rcsb.org/pdb/home/home.do).

## Abbreviations

HT1: Hereditary tyrosinemia type 1
FAH: Fumarylacetoacetate hydrolase
HSF: Human Splicing Finder
SA: Succinylacetone
NTBC: Nitisinone
HPPD: Parahydroxyphenylpyruvic acid dioxygenase
kb: Kilobase
PDB: Protein Data Bank
ALT: Alanine transaminase
AST: Aspartate transaminase
GGT: Gamma glutamyl transferase
AFP: Alpha-fetoprotein
RI: Reference interval
